# Emotional and behavioral problems and associated factors among children and adolescents on highly active anti-retroviral therapy in public hospitals of West Gojjam zone, Amhara regional state of Ethiopia, 2018: a cross-sectional study

**DOI:** 10.1186/s12887-019-1453-3

**Published:** 2019-05-03

**Authors:** Demewoz Kefale, Abdisa Boka, Zureyash Mengstu, Zelalem Belayneh, Shegaw Zeleke

**Affiliations:** 1 Department of Pediatrics and Child Health Nursing, College of Health Science, Debre Tabor University, Debre Tabor, Ethiopia; 2 School of Nursing and Midwifery, College of Health Science, Addis Ababa University, Addis Ababa, Ethiopia; 3 Department of Psychiatry, College of Health and Medical Science, Dilla University, Dilla, Ethiopia

**Keywords:** Behavioral and emotional problems, Pediatric symptomatology check list, Children and adolescent, HIV/AIDS and mental health, West Gojjam

## Abstract

**Background:**

Children and adolescents with HIV/AIDS are more likely to have emotional and behavioral problems than the general population. This can result in a continuing negative influence on the quality of life, school performance, immunity and co-morbidity of children and adolescents with HIV/AIDS.

**Objective:**

To assess the prevalence and associated factors of Emotional and Behavioral Problems among children and adolescents on Highly Active Anti-Retroviral Therapy in the public hospitals of West Gojjam Zone, Amhara regional state of Ethiopia.

**Methods:**

An institutional based cross sectional study was conducted by screening 411 children and adolescents for emotional and behavioral problems using Pediatric Symptomatology Check List (PSCL). Systematic random sampling technique was used to select the study participants. Data analysis was done using SPSS version 23. Bivariable and multivariable logistic regression analysis were fitted to identify factors associated with Emotional and Behavioral Problems. Odds ratio (OR) with 95% confidence interval (CI) was computed to determine the level of significance.

**Result:**

Out of the total 411 participants, 43.6% were screened positive for Emotional and Behavioral Problems. Lower age (AOR = 5.33, 95%CI: 2.56–11.04), having non-kin care giver (AOR = 4.64, 95%CI: 1.20–17.90), parental loss (AOR = 2.15, 95%CI: 1.03–4.49), non self -disclosure of HIV sero status (AOR = 1.99, 95% CI: 1.16–3.41) and having distressed care giver (AOR = 1.64, 95%CI: 1.04–2.57) had statistically significant association with EBPs.

**Conclusion:**

The prevalence of Emotional and Behavioral Problems is high among children and adolescents on HAART. Lower age, care giver’s mental distress, non-self disclosure status, having non-kin care giver and parental loss were variables significantly associated with EBPs. This demonstrates a need for the integration of Mental Health and Psycho Social Support (MHPSS) service with HIV/AIDS care.

**Electronic supplementary material:**

The online version of this article (10.1186/s12887-019-1453-3) contains supplementary material, which is available to authorized users.

## Background

Globally, more than 3.4 million children and adolescents are living with HIV or died due to AIDS related cases [1]. Additionally, an estimated 29 adolescents acquired HIV every hour. Childhood and adolescence is the only age group in which AIDS-related deaths are not decreasing [2] as being adolescent by itself is a risk for the vulnerability of HIV infection [3]. Chronic illness such as HIV/AIDS often represents a traumatic change in the life of children and adolescents which significantly affects their health, welfare, social, mental and economic development [4].

There is a world wide effort to provide sustainable antiretroviral medication to prolong life and to reduce stigma. This calls a more holistic and comprehensive approach to HIV care and treatment [5, 6]. However, HIV treatment outcome of children and adolescents has not yet been satisfactory [7] due to the co-morbidity [8] and un usual onset of Emotional and Behavioral Problems (EBPs) [9].

Health related factors clearly play a role in emotional and behavioral problems. HIV is an illness that affects emotions and behaviors of children and adolescents [10–12]. This can accelerate AIDS-related mortality among HIV-positive children and adolescents regardless of the scale up of antiretroviral therapy innovations [13–15]. It can also have diverse and devastating consequences like suicidal ideation and attempt, school absenteeism and high drop-out rates, high rates of grade retention, engagement in risky behavior and being hyperactive (16), and may progress to other primary psychiatric disorders like conduct disorder, antisocial personality disorder, anxiety, depression and others [12].

It is also recommended that HIV/AIDS care service is expected to have a holistic approach applicable to all children and adolescents affected by AIDS in all settings [16, 17]. In contrast to this, health-care systems in low income and middle income countries have not adequately addressed the identification and interventions of EBPs in children and adolescents with AIDS. Moreover, the focus of most other studies was on adults’ health and little emphasis is given for children and adolescent’s health [18–20]**.** The low level of awareness about emotional and behavioral symptoms [21], the unusual nature of the symptoms, the wide spread traditional explanatory models and preference of traditional treatment options might have significant challenges upon the utilization of mental health and psychosocial support services for children and adolescents with HIV/AIDS [22].

Although there is a study done in Ethiopia reporting about the EBPs of children and adolescents on HAART, it is limited to Addis Ababa (the capital city of Ethiopia), and did not address the other parts of Ethiopia [23]. This sounds a need to assess the EBPs of children and adolescents in rural parts of Ethiopia which have different cultural context and living standards from Addis Ababa.

## Methods

### Study design, period and setting

An institutional based cross-sectional study was conducted in West Gojjam zone public hospitals from March 30/2018 to May30/2018. West Gojjam zone is found in Amhara Region States of Ethiopia and its zonal city is Finote Selam which is found 330 km North West from Addis Ababa. The zone has 6 public hospitals and only five of them provide ART service for about 3, 214 children and adolescents.

### Sample size calculation and sampling procedure

The assumptions made for sample size calculation were a 95% confidence interval, and 50% expected prevalence EBPs to get the maximum sample size and a 5% margin of error. By adding a 10% non-response rate, the total sample size was 423. Systematic random sampling was employed to select the study participants. Initially, the total expected number of children and adolescents attending ART clinic during the study period was calculated from the records of each hospital. Then, the number of children and adolescents included in each hospital was determined based on the proportionate population size. The sampling interval (K) was determined by dividing the total number of children and adolescents on HAART in each hospital to the sample size to be drawn from that hospital. Lottery method was used to select the first participant between one and K. Subsequently, K value was added until the sample size allocated to each hospital was reached.

### Data collection instrument and techniques

An interviewer administered questionnaire was used for the data collection (Additional file 1). The questionnaire was prepared in English and translated in to Amharic (the commonly spoken language in the study area), and finally back to English to test the accuracy of translation. The Amharic version questionnaire was pre-tested on 22 (5%) of children and adolescents on HAART at Felegehiwot Hospital (not included in the study). The questionnaire had five parts including sociodemographic characteristics, clinical related factors, caregiver related factors, Pediatric Symptomatology Check list (PSCL) and Self Reporting Questionnaire (SRQ-20).

Parents/care givers of children and adolescents who fulfilled the inclusion criteria (age range of 5–18 years, having at least for 1 month follow up and never been admitted at inpatient care) were requested to give assent on the behalf of their child or adolescent after brief explanation about the scope and objectives of the study.

The PSCL was used to screen Emotional and Behavioral Problems among children and adolescents on HAART. It is a tool validated to assess emotional and behavioral problems in children and adolescents with HIV infection and other chronic diseases [24–26]. PSCL consists of a set of 35 questions with three possible responses rated as never (0), sometimes (1), often (2).. The total score was calculated by adding together the scores for each of the 35 questions and had a range scores from 0 to 70. A cut off point of 24 and above for age 5–6 years and 28 and higher for age > 6 years were taken to indicate EBPs [27]. The symptoms of PSCL was translated and modified in a culturally acceptable way.

Care givers’ mental distress was measured using WHO’s Self Reporting Questionnaire (SRQ-20) assessment tool [28]. SRQ has 20 “Yes” or “No” questions with a total scores of 0 to 20. A score of 7 and above was considered as a cutoff point of having mental distress [10].

The data was collected by eight BSc (Bachelor of Science) level health professionals with the supervision of two MSC health professionals after two consecutive days of training. During the interview, caregivers were also participated to answer some questions when the child had difficulty of answering questions adequately. Finally, some clinical related data were extracted from the hospital records.

### Operational definition

Non self-disclosure of HIV sero status: is defined as the child or adolescent who were not aware of his/her HIV positive sero-status status because the professional or the care giver did not inform as he/she is HIV sero-positive.

### Data analysis and interpretation

The collected data were checked for its completeness and consistency. Then, it was coded and entered in to the computer using EP-data version 4.2software and transformed in to SPSS version 23 for analysis. Descriptive statistics was carried out to measure the magnitude and distributions of EBP and the result was presented using text and tables. Both bivariable and multivariable logistic regression analysis were fitted to identify factors associated with emotional and behavioral problems. Variables with a *P*-value of less than 0.25 in the bivariable analysis were transformed into the multivariable analysis. In multivariable analysis, variables with a *p*-value of less than 0.05 were considered statistically significant. Adjusted odds ratio (AOR) with the corresponding 95% confidence interval (CI) was used to show the strength of association.

## Results

### Socio-demographic characteristics of children and adolescents on HAART in public hospitals of west Gojjam zone

A total of 411 respondents participated in the study with a response rate 97.2%. The mean age (±SD) of respondent was 11.67 (±3.25) years. More than half, (59.9%), of them were males. Regarding their residency, 67.2% were from urban. (Table 1).

**Table 1:** Socio-demographic characteristics of children and adolescents on HAART in West Gojjam Zone Public Hospitals, North West Ethiopia, 2018(*N* = 411)

Variables	Categories	Frequency	Percentage
Ages (in years)	5–9	110	26.8
	10–14	211	51.3
Sex	Male	246	59.9
	Female	165	40.1
Religion	Orthodox	352	85.6
	Protestant	42	10.2
	Muslim	17	4.1
Residency	Urban	276	67.2
	Rural	135	32.8
School attendance	Yes	335	81.5
	No	76	18.5
Family size	1–3	232	56.4
	4–6	156	38.0
	> 6	23	5.6
Family monthly income	< 1660.34	264	59.9
	> 1660.34	165	40.1

*Abbreviations: HAART* Highly Active Anti Retroviral Therapy

### Clinical related characteristics

About 84.4% were on ART treatment for more than 6 months duration, and 70.1% knew their HIV status (Table 2).

**Table 2:** Clinical related characteristics of children and adolescents on HAART in West Gojjam Zone Public Hospitals, North West Ethiopia, 2018, (*N* = 411)

Variables	Categories	Frequency	Percentage
Duration of ART treatment	1–6 months	64	15.6
	> 6 months	347	84.4
Self Disclosure of HIV sero-status	Disclosed	288	70.1
	Not disclosed	123	29.9
Recent CD4 count	< 200 cells/mm^3^	6	1.5
	200–349 cells/mm^3^	29	7.1
	350–500 cells/mm^3^	66	16.1
	> 500 cells/mm^3^	310	75.4

*Abbreviations: HAART* Highly Active Anti Retroviral Therapy

### Care-giver related characteristics

Almost half, 48.7% (*n* = 200), of the care givers of children and adolescents were College/Higher educational level and 83.0% (*n* = 341), of care-givers were child’s own parents. About 44.0% (*n* = 181), had single parental loss which mean that either father or mother is died. Most, 79.3% (*n* = 326), of care givers of children and adolescents had positive HIV sero- status. Additionally, about 41.8% of the care givers were screened positive for mental distress.

### Distributions of symptoms of behavioral and emotional problems

“Seems to be having less fun” and “School grades dropping” are the most commonly displayed symptom endorsed by 31.1 and 29.8% of children and adolescents on HAART, respectively However “Acts as if driven by a motor” and “Takes unnecessary risks” are the least commonly symptoms mentioned by 0.8% of the respondents (Table 3).

**Table 3:** Distributions of Pediatrics Symptomatic Check List items among children and adolescents on HAART in West Gojjam Zone Public Hospitals, North West Ethiopia, 2018 (*N* = 411)

Symptoms	Responses		
	Never(0) N/%	Sometimes(1)N/%	Often(2)N/%
Complains of aches and pains	315/76.6%	37/9%	59/14.4%
Spends more time alone	359/87.3%	29/7%	23/5.7%
Tires easily, has little energy	341/83%	57/13.8%	13/3.2%
Fidgety, unable to sit still	318/77.4%	35/8.5%	58/14.1%
Has trouble with teacher	309/75.1%	67/16.3%	35/8.6%
Less interested in school	293/71.2%	89/21.6%	29/7.2%
Acts as if driven by a motor	397/96.5%	11/2.7%	3/0.8%%
Daydreams to much	380/92.4%	20/4.8%	11/2.8%
Distracted easily	243/59.1%	112/27.2%	56/13.7%
Is afraid of new situations	293/71.2%	95/23.1%	23/5.7%
Feels sad, unhappy	225/54.7%	125/30.4%	61/14.9%
Is irritable, angry	215/52.3%	84/20.4%	112/27.3%
Feels hopeless	345/83.9%	34/8.2%	32/7.9%
Has trouble concentrating	276/67.1%	54/13.1%	81/19.8%
Less interested in friends	338/82.2%	17/4.1%	56/13.8%
Fights with other children	189/45.9%	151/36.7%	71/17.4%
Absent from school	316/76.8	53/12.8%	42/10.4%
School grades dropping	32/7.7%	257/62.5%	122/29.8%
Is down on him or herself	376/91.4%	23/5.6%	12/3%
Visits the doctor with nothing wrong	284/69%	51/12.4%	76/18.6%
Has trouble sleeping	300/72.9%	72/17.5%	39/9.6%
Worries a lot	379/92.2%	21/5.1%	11/2.7%
Wants to be with you more than before	372/90.5%	9/2.1%	30/8.4%
Feels he or she is bad	359/87.3%	23/5.5%	29/7.2%
Takes unnecessary risks	401/97.5%	7/1.7%	3/0.8%
Gets hurt frequently	334/81.2%	26/6.3%	51/12.5%
Seems to be having less fun	121/29.4%	138/33.5%	152/31.1%
Acts younger than children his or her age	403/98%	5/1.2%	3/0.8%
Does not listen to rules	303/73.7	63/15.3%	45/11%
Does not show feelings	399/97%	5/1.2%	7/1.7%
Does not understand other’s feelings	318/77.3%	12/2.9%	81/19.8%
Teases other	382/92.9%	7/1.7%	22/5.4%
Blames others for his or her troubles	262/63.7%	132/32.2%	17/4.1%
Takes things that do not belong to him/her	325/79%	32/7.7%	54/13.3%
Refuses to share	63/15.3%	121/29.4%	7/1.7%

*Abbreviations: HAART* Highly Active Anti Retroviral Therapy

### Prevalence and factors associated with emotional and behavioral problems

Out of the total 411 children and adolescents participated in the study, 43.6% (*N* = 179) were screened positive for EBPs with CI = (38.8–48.4). Bivariable and multivariable logistics analysis were computed to identify factors associated with EBPs. In the multivariable logistic analysis, age ranges of 5–9 years, parental loss, non-self disclosure to HIV-Positive sero status, having non kin care-givers and care giver’s mental distress had statically significant association with EBPs (Table 4).

**Table 4:** Bivariable and multivariable analysis of Emotional and Behavioral Problems among children and adolescent on HAART in West Gojjam Zone Public Hospitals, North West Ethiopia, 2018(*N* = 411)

Variables	Categories	EBPs status		COR(95%CI)	AOR(95%CI)	*P*-Value
		Positive N (%)	Negative N (%)			
Age in years	5–9	70 (63.6)	40 (36.4)	3.88 (2.14–7.00)	5.33 (2.56,11.04)***	0.00
	10–14	81 (38.4)	130 (61.6)	1.38 (0.82–2.33)	1.95 (1.08–3.55*	0.03
	15–18	28 (31.1)	62 (68.9)	1.00	1.00	
School attendance	Attended	137 (40.9)	198 (59.1)	1.00	1.00	
	Non attended	42 (55.3)	34 (44.7)	1.79 (1.08–2.95)	0.61 (0.31–1.18)	0.14
Caregiver’s educational level	College	95 (47.5)	105 (52.5)	1.00	1.00	
	Secondary	14 (63.6)	8 (36.4)	1.93 (0.78–4.81)	0.81 (0.26–2.48)	0.71
	Primary	13 (40.6)	19 (59.4)	0.76 (0.35–1.61)	0.99 (0.43–2.33)	0.99
	Illiterate	57 (36.3)	100 (63.7)	0.63 (0.41–0.97)	0.64 (0.40–1.04)	0.07
Caregiver’s relationship	Parent	135 (39.6)	206 (60.4)	1.00	1.00	
	Relative	35 (61.4)	22 (38.6)	2.43 (1.37–4.32)	1.95 (0.93–4.07)	0.08
	Non-kin	9 (69.2)	4 (30.8)	3.43 (1.04,11.37)	4.64 (1.20–17.90)*	0.03
Parental loss	Both alive	56 (33.9)	109 (66.1)	1.00	1.00	
	Single	84 (46.4)	97 (53.6)	1.69 (1.09–2.60)	1.73 (1.07–2.81)*	0.03
	Both died	39 (60.0)	26 (40.0)	2.92 (1.62–5.28)	2.15 (1.03–4.49)*	0.04
HIV self disclosure status	Disclosed	104 (36.1)	184 (63.9)	1.00	1.00	
	Non disclosed	75 (60.98)	48 (39.02)	2.76 (1.79–4.27)	1.99 (1.16–3.41)**	0.01
Care-giver’s mental distress	Distressed	87 (50.6)	85 (49.4)	1.64 (1.10–2.43)	1.64 (1.04–2.57)*	0.03
	Not distressed	92 (38.5)	147 (61.5)	1.00	1.00	

*Abbreviations: HAART* Highly Active Anti Retroviral Therapy

*P-value< 0.05, **p < 0.01, ***p < 0.001

## Discussion

Findings of the current study showed that the prevalence of emotional and behavioral problems among children and adolescents on HAART in West Gojjam Zone public hospitals was 43.6% with CI = (38.8–48.4). This finding is in line other similar studies of India (40%) [29] and Ethiopia (39.3%) [23]. However, it shows a lower result than a study conducted in Uganda (58.5%) [30]. This might be due to the difference of screening tools used [10, 11]. But it was higher than a study conducted in USA (7.4%) [31], India (30%) [11, 27] and Portugal (26.7%) [32]**.** This variation might be explained due to the fact that there is a great difference in the socio-economical, health care delivery system and cultural context of the study subjects [7, 33].

The findings of the current study showed a higher prevalence of EBPs than other similar studies done in Ambo district (17.7%) [34] and Butajira district (6.5%) [35] among non-HIV infected children. This clearly showed that the prevalence of emotional and behavioral problems among children and adolescents with HIV/AIDS is higher than similar groups in the general population. This higher prevalence might be due to direct viral effects on the brain, poor self-esteem, stigma and discrimination [36] and pill burdens [37] of children and adolescents with HIV/AIDS. The odds of developing emotional and behavioral problems among children and adolescents who were not aware of their HIV sero positive status was 1.99 times (AOR = 1.99, 95%CI: 1.16, 3.41) higher than children and adolescents who were aware their HIV sero positive status. This is consistent with other similar studies done in Malawi [19] and South Africa [3] and reported by WHO [38]. This could be due to the fact that children and adolescents who did not recognize their HIV sero positive status may have confusion/tension why they have to continue to take drugs every day and maintain false understanding/perception of the illness [38].

The odds of having emotional and behavioral problems among children and adolescents who had non-kin care givers was 4.64 times (AOR = 4.64, 95%CI: 1.20–17.90) higher as compared children and adolescents whose care givers are child’s own parents. This idea is consistent with previous studies of USA [39]**,**Thailand [40], India [27], South Nigeria [22] and Kenya) [41]. The possible explanation might be child’s own parents can provide a more protective and parenthood care than non-kin care givers, and children and adolescents can have good attachment with individuals whom they attached to earlier [18].

Similarly, children and adolescents who had lost both parents had 2.15times increased odds of having emotional and behavioral problems as compared to children and adolescents whose parents are alive. This idea is similar with study done in Malawi [19], South Africa [3, 42], Nigeria [22] and Kenya [41]. The possible reason might be due to the difficulty of children and adolescents to adapt themselves to live without parents which may pose them to a prolonged mental and behavioral problems [8].

Studies from different parts of the world (USA [39, 43], London UK [44], and South Africa [42]**)** identified that care givers’ mental distress has significant association with EBPs of children and adolescents with HIV/AIDS. The findings of the current study also confirmed that mental distress can increase the odds of having EBPs by1.64 times among children and adolescents with HIV/AIDS This possible reason might be due to the fact that there is a possibility of displaying “Expressed Emotion” towards children and adolescents and negligence to take care when they become psychologically distressed [42].

### Limitation of the study

This study has some limitations. First, PSCL is a screening tool for EBPs and the diagnosis of EBPs never been confirmed. This may overestimate the prevalence of EBPs. Second**,** the PSCL tool is not validated in Ethiopia**.** Third, when the child/adolescent had difficulty in answering questions, care givers participated on their child’s behalf. This might not be consistent with what the child would have answered. Fourth, family monthly income was assessed simply by asking the estimated amount of money they earned every month. It would have been more accurate to use the wealth-index. Finally, the cross-sectional nature of the study might not show the direct cause and effect relationships of variables.

## Conclusion

The prevalence of Emotional and Behavioral Problems is high among children and adolescents on HAART. Lower age, care giver’s mental distress, non self-disclosure status, having non-kin care giver and parental loss were variables significantly associated with EBPs. This demonstrates a need for the integration of Mental Health and Psycho Social Support (MHPSS) service with HIV/AIDS care. Moreover, disclosing their HIV sero status, providing care with kins/own parents and, prevention and treatment of care giver’s mental distress are very crucial too.

## Additional file

Additional file 1: English version questionnaire. (DOCX 19 kb)

## Abbreviations

AIDS: Acquired Immune Deficiency Syndrome
AOR: Adjusted Odds Ratio
ART: Antiretroviral Therapy
CI: Confidence Interval
COR: Crude Odd Ratio
EBP: Emotional and Behavioral Problems
FMOH: Federal Ministry of Health
HAART: Highly Active Antiretroviral Therapy
HIV: Human Immune Virus
MHPSS: Mental Health and Psycho Social Support
PLWHA: People Living with HIV/AIDS
PSCL: Pediatrics Symptomatology Check List
SPSS: Statistical package for social sciences
SRQ: Self Response Questionnaire
