# Supplementary material for: Emotional and behavioral problems and associated factors among children and adolescents on highly active anti-retroviral therapy in public hospitals of West Gojjam zone, Amhara regional state of Ethiopia, 2018: a cross-sectional study
Source: BMC Pediatr. 2019 May 3;19:141. doi: 10.1186/s12887-019-1453-3 (PMC6498648; doi:10.1186/s12887-019-1453-3)
Supplement: Supplementary file 1 — English version questionnaire. (DOCX 19 kb) [file 12887_2019_1453_MOESM1_ESM.docx]

# ENGLISH VERSION QUESTIONNAIRE CIRCLING THE ALTERNATIVE

### INFORMATION SHEET

Good morning/good afternoon! My name is . I am here today to collect data on emotional and behavioral problems and associated factors among children and adolescents on highly active antiretroviral therapy at governmental public health institutions, West Gojjam zone.

The study is being conducted by Demewoz kefale, postgraduate program student at Addis Ababa University College of health sciences school of Allied health, Department of Nursing and Midwifery. The objective of this study is to assess the prevalence and associated factor of emotional and behavioral problems among children and adolescents on HAART.

You are being asked to take part in this study and to respond genuinely. Your name will not be written in this form and will never be used in connection with any information you tell us.

There is no possible risk associated with participating in this study except the time spent for completing the questionnaire. All information given by you will be kept strictly confidential. Your participation is voluntary and you are not obligated to answer any question you do not wish to answer. If you feel discomfort with the question, it is your right to withdraw at any time you wish. If you have questions regarding this study or would like to be informed of the results after its completion, please feel free to contact the principal investigator.

Address of the principal investigator:

Cell phone: +251920253332, Email- [demewozk@yahoo.com](mailto:demewozk@yahoo.com)

Data collector’s name Signature Date

### SOCIO-DEMOGRAPHIC CHARACTERISTIC QUESTIONNAIRE

**I will ask some question about your child’s socio demographic charactostics.**

1. Age in years -------------------

1. Sex A. Male B. Female
2. Residence A. urban B. rural
3. Religion A. Orthodox B. protestant C. Muslim D. catholic E. Other
4. School Attendance A. yes B. no
5. Care giver educational level A. 1-11^th^ grade B.12th completed C. College/ Higher D. Illiterate
6. Care –giver’s relationship with children A. parent B. relatives C. others specify-----
7. Parental loss A. single B. double C. no (both alive)
8. Family size A. 1-3 B. 4-6 C.>6 D. other
9. Income level (ETB)……………………………..……….

**Clininical related characteristics**

1. Disclosure to self of HIV sero-status A. yes B. no

2. Care giver’s HIV status A. positive B. Negative C. unknown

1. Did your child diagnosis for mental illness before? A. yes B. no
2. Treatment duration on ART A.1-6 months B. 6≥ months
3. Number of recent CD4 Cells (cells/mm3) of your child………........

#### SELF-REPORTING QUESTIONNAIRE (SRQ-20) (care-giver distress)

#### Here, I am going to ask you some question about you not about the child. Please say “Yes” or “ No” for these questions.

|  | YES | **NO** |
| --- | --- | --- |
| 1. Do you often have headaches? |  |  |
| 2. Is your appetite poor? |  |  |
| 3. Do you sleep badly? |  |  |
| 4. Are you easily frightened? |  |  |
| 5. Do your hands shake? |  |  |
| 6. Do you feel nervous, tense or worried? |  |  |
| 7. Is your digestion poor? |  |  |
| 8. Do you have trouble thinking clearly? |  |  |
| 9. Do you feel unhappy? |  |  |
| 10. Do you cry more than usual? |  |  |
| 11. Do you find it difficult to enjoy your daily activities? |  |  |
| 12. Do you find it difficult to make decisions? |  |  |
| 13. Is your daily work suffering? |  |  |
| 14. Are you unable to play a useful part in life? |  |  |
| 15. Have you lost interest in things? |  |  |
| 16. Do you feel that you are a worthless person? |  |  |
| 17. Has the thought of ending your life been on your mind |  |  |
| 18. Do you feel tired all the time? |  |  |
| 19. Are you easily tired |  |  |
| 20. Do you have uncomfortable feelings in your stomach? |  |  |
| SRQ-20 Total Score (total of yes) |  |  |

**PEDIATRIC SYMPTOMS CHECKLIST (PSCL)**

Please tell me the heading that best describes your child’s condition

|  | Symptoms | NEVER (0) | SOMETIMES (1) | OFTEN (2) |
| --- | --- | --- | --- | --- |
| 1 | Complains of aches and pains |  |  |  |
| 2 | Spends more time alone |  |  |  |
| 3 | Tires easily, has little energy |  |  |  |
| 4 | Fidgety, unable to sit still |  |  |  |
| 5 | Has trouble with teacher |  |  |  |
| 6 | Less interested in school |  |  |  |
| 7 | Acts as if driven by a motor |  |  |  |
| 8 | Daydreams to much |  |  |  |
| 9 | Distracted easily |  |  |  |
| 10 | Is afraid of new situations |  |  |  |
| 11 | Feels sad, unhappy |  |  |  |
| 12 | Is irritable, angry |  |  |  |
| 13 | Feels hopeless |  |  |  |
| 14 | Has trouble concentrating |  |  |  |
| 15 | Less interested in friends |  |  |  |
| 16 | Fights with other children |  |  |  |
| 17 | Absent from school |  |  |  |
| 18 | School grades dropping |  |  |  |
| 19 | Is down on him or herself |  |  |  |
| 20 | Visits the doctor with nothing wrong |  |  |  |
| 21 | Has trouble sleeping |  |  |  |
| 22 | Worries a lot |  |  |  |
| 23 | Wants to be with you more than before |  |  |  |
| 24 | Feels he or she is bad |  |  |  |
| 25 | Takes unnecessary risks |  |  |  |
| 26 | Gets hurt frequently |  |  |  |
| 27 | Seems to be having less fun |  |  |  |
| 28 | Acts younger than children his or her age |  |  |  |
| 29 | Does not listen to rules |  |  |  |
| 30 | Does not show feelings |  |  |  |
| 31 | Does not understand other’s feelings |  |  |  |
| 32 | Teases other |  |  |  |
| 33 | Blames others for his or her troubles |  |  |  |
| 34 | Takes things that do not belong to him/her |  |  |  |
| 35 | Refuses to share |  |  |  |

#### Thank you so much for your cooperation
